# An Acknowledgment

**Published:** 1853-12

**Authors:** 


An Acknowledgement.—We are happy to be able to record the donation of a valuable and tasteful collection of Botanical specimens, to the Medical Museum, by Mr. R. W. Pitman, of West Point, Iowa. The numerous array of pressed flowers alluded to, was evidently prepared and arranged with much care, and our generous friend has our grateful thanks for his kindly remembrance of the Institution. We are pleased at so early and acceptable a response to our recent "Invitation," and hope many others, friends of the Medical College, in various parts of the State, may be prompted to go and do likewise.

